# IMPACT OF HEALTHY LIFESTYLE FACTORS ON LIFE EXPECTANCY AND LIFETIME HEALTHCARE COST: A NATIONWIDE COHORT STUDY

**DOI:** 10.1093/geroni/igad104.3414

**Published:** 2023-12-21

**Authors:** Wei-Cheng Lo, Tsuey-Hwa Hu, Cheng-Yu Shih, Hsien-Ho Lin, Jing-Shiang Hwang

**Affiliations:** Taipei Medical University, New Taipei City, New Taipei, Taiwan (Republic of China); Academia Sinica, Taipei, Taipei, Taiwan (Republic of China); National Taiwan University, Taipei, Taipei, Taiwan (Republic of China); National Taiwan University, Taipei, Taipei, Taiwan (Republic of China); Academia Sinica, Taipei, Taipei, Taiwan (Republic of China)

## Abstract

In an aging society with increasing life expectancy, the significance of healthy behaviors has garnered considerable attention. Research indicates that adopting healthy behaviors not only contributes to a longer life expectancy but also leads to reduced healthcare expenditure. Our study aims to investigate the impact of individual and combined healthy lifestyle factors on life expectancy and lifetime healthcare expenditure in Taiwan. Analyzing data from the National Health Interview Survey cohort, we examined five healthy lifestyle behaviors: nonsmoking, avoiding excessive alcohol consumption, regular physical activity, sufficient fruit and vegetable intake, and maintaining a normal weight. By employing a rolling extrapolation algorithm, we estimated the lifetime survival function of the study cohorts and calculated the life expectancy and lifetime healthcare expenditure of populations with and without healthy lifestyle factors. Our analysis of 19,893 participants aged 30 and above revealed that adopting a healthy lifestyle is associated with longer life expectancy and reduced healthcare expenditure in Taiwanese adults. Participants adhering to all five behaviors gained 11.93 years in life expectancy compared to those with none, with an annual average healthcare expenditure reduction of $7,308. Smoking and diet were the most significant risk factors for shorter life expectancy, with current and former smokers experiencing a 4.02-year reduction and individuals with sufficient fruit and vegetable intake gaining 3.69 years compared to those with insufficient intake. These findings offer a comprehensive understanding of the impact of healthy lifestyle factors on overall health and economic burden, providing evidence-based guidance for policymakers and stakeholders in promoting healthy aging.

